# Involvement of eIF6 in external mechanical stretch–mediated murine dermal fibroblast function via TGF-β1 pathway

**DOI:** 10.1038/srep36075

**Published:** 2016-11-08

**Authors:** Qin Shu, Jianglin Tan, Van Daele Ulrike, Xiaorong Zhang, Jiacai Yang, Sisi Yang, Xiaohong Hu, Weifeng He, Gaoxing Luo, Jun Wu

**Affiliations:** 1 Institute of Burn Research, State Key Laboratory of Trauma, Burn and Combined Injury, Southwest Hospital, The Third Military Medical University, Chongqing, 400038, People’s Republic of China; 2 Nursing Department, The Third Military Medical University, Chongqing, 400038, People’s Republic of China; 3 Chongqing Key Laboratory for Disease Proteomics, The Third Military Medical University, Chongqing, 400038, China; 4 Department of Rehabilitation Sciences and Physiotherapy, University of Antwerp, Belgium; 5 Department of Urology, Second Affiliated Hospital, Third Military Medical University, Chongqing, 400037, People’s Republic of China

## Abstract

External mechanical loading on a wound commonly increases fibrosis. Transforming growth factor-β1 (TGF-β1) has been implicated in fibrosis in various models, including the mechanical force model. However, the underlying mechanism is unclear. Our previous experiments suggested that eukaryotic initiation factor 6 (eIF6) acted as a regulator of TGF-β1 expression, and negatively impact on collagen synthesis. Our current results showed that external mechanical stretching significantly increased COL1A1, TGF-β1 and eIF6 expression as well as dermal fibroblasts proliferation, both *in vitro* and *in vivo*. eIF6 –deficient (eIF6^+/−^) cells exhibited significantly higher levels of COL1A1, and these levels increased further with external mechanical stretching, suggesting that mechanical stretching plays a synergistic role in promoting COL1A1 expression in eIF6^+/−^ cells. Inhibition of TGFβR I/II by LY2109761 decreased COL1A1 protein expression in eIF6^+/−^ dermal fibroblasts in a cell stretching model, and attenuated granulation tissue formation in partial thickness wounds of eIF6^+/−^ mice. These data suggest that mechanical stretching has a synergistic role in the expression of COL1A1 in eIF6^+/−^ cells, and is mediated by activation of TGFβRI/II. Taken together, our results indicate that eIF6 may be involved in external mechanical force-mediated murine dermal fibroblast function at least partly through the TGF-β1 pathway.

Pathological scarring, including hypertrophic and keloid scarring, is a common and clinically significant complication after severe burn injuries^1^,^2^,^3^. The matrix proteins secreted by hypertrophic scar fibroblasts differ from those secreted by normal skin cells^4^. The extra cellular matrix (ECM) in hypertrophic scars never achieves the flexibility or strength of the original tissue^5^, often leading to physical dysfunction and psychological stress^2^. At present, the mechanisms underlying hypertrophic scar formation are not fully understood.

Mechanical loading profoundly influences the composition and structure of the ECM^6^. Many *in vivo* studies have shown that keloid and hypertrophic scars have few cellular abnormalities and often occur at sites that are constantly or frequently subjected to external mechanical skin stretching. Tension during healing appears to provoke an excessive or deranged response, resulting in fibrogenesis and increased deposition of collagen^7^,^8^. This response suggests that mechanosensory pathways may be responsible for mechanical force-mediated fibrogenesis^8^.

The structure and function of dermal fibroblasts, the major cell type involved in wound repair, are affected by mechanical loading. Local mechanical forces within an open wound subject dermal fibroblasts to internal forces, either by extending their membranes or by rearranging their actin cytoskeletons^9^. In this situation, characterized by increased α-smooth muscle actin (α-SMA) expression, collagen synthesis and contraction, an abnormally large number of dermal fibroblasts differentiate into myofibroblasts^9^. Transforming growth factor-β (TGF-β) is one of the most important molecules contributing to the activation and maintenance of myofibroblasts^10^. In homeostatic conditions, TGF-β is sequestered in the ECM as part of the large latent complex (LLC), which includes latency-associated peptides (LAPs) and latent TGF-β-binding proteins (LTBPs)^11^. Upon injury, the binding of integrins to LTBPs, together with increased mechanical forces, can cause the release of TGF-β from the LLC, allowing it to interact with two type I and two type II receptors and subsequently with phosphorylated Smad proteins^12^,^13^, resulting in the formation of a hypertrophic scar. Cyclic mechanical strain results in a significant increase in active TGF-β1 levels, leading to a p-Smad2-mediated increase in the transcription of downstream regulatory factors^14^. These observations suggest that the activity of TGF-β1 plays an important role in the formation of hypertrophic scars under external mechanical loading, and that the identification of a TGF-β1 inhibitory factor will be a key step in the research for treatments to control hypertrophic scarring.

Recently, eukaryotic initiation factor 6 (eIF6), also known as protein p27BBP (beta 4 binding protein), has been identified as a regulator of TGF-β1 expression and myofibroblast differentiation. eIF6 is also involved in the development of hypertrophic scars, and is therefore a potential target for the control of hypertrophic scarring^15^. Our earlier study showed that eIF6 regulates TGF-β1 expression at the transcriptional level by facilitating occupancy of the TGF-β1 promoter by Sp1 rather than H2A.Z^16^. *In vivo* studies also show that a heterozygous eIF6^+/−^ mouse exhibits enhanced TGF-β1 production after skin injury, coupled with increased numbers of α-smooth muscle actin (α-SMA)^+^ myofibroblasts^16^. We hypothesized that eIF6 plays a role in maintaining fibroblast homeostasis under external mechanical loading through TGF-β pathway regulation. The eIF6 protein has five quasi-identical α/β subdomains^17^ that link integrin a6β4 to the intermediate filament cytoskeleton and form a multivalent laminin receptor^18^. These findings suggest to us that eIF6 may be a mechano-sensitive protein, essential for cytoskeletal integrity.

In this study, we examined the expression of eIF6 and components of TGF-β/Smad signaling pathway in cell stretch and dermal stretch models to determine their effects on murine dermal fibroblasts. Our results shed light on a possible role for eIF6 and TGFβR I/II in external mechanical force-mediated dermal fibroblast function and fibrosis, and may lead to new strategies for the prevention and/or treatment of pathological scarring in humans.

## Results

### The effect of mechanical stretch on eIF6 expression *in vivo* and *in vitro*

**Figure 1:**
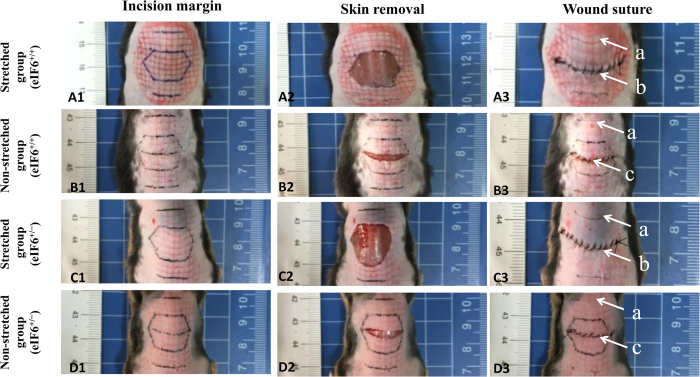
Development of an external mechanical stretch model using partial-thickness back skin wounds in mice. (A1–3, C1–3) STRETCHED group: Photographs of the partial-thickness wounds with grid markings before and after mechanical stretching. The partial-thickness wounds are marked as arrow a. The distance between the partial-thickness wound and the suture line (arrow b) is 0.6 cm. Sustained mechanical stretching was generated using a skin defect (a hexagonally shaped skin resection) and tension suturing (edge stitching), as indicated by arrow b. The grid was used to characterize skin deformation induced by the stretching. (B1–3, D1–3). NON-STRETCHED group: Photographs of partial-thickness wounds with grid markings before and after a sham operation, followed by suturing without the introduction of stretching (indicated by arrow c). n = 4 for each group.

**Figure 2:**
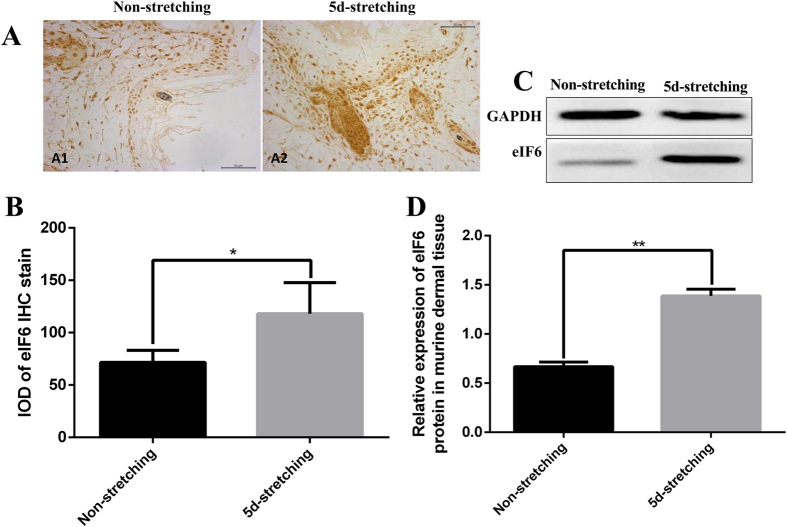
Expression of eIF6 in wild type murine dermis subjected to external mechanical stretching. Non-stretched partial-thickness wounds were compared with stretched wounds in wild type mice 5 days after suturing. (**A**) Immunohistochemical (IHC) staining of eIF6 in newly formed granulation tissue (eIF6 is stained brown; 400× magnification). (**B**) Integral optical density (IOD) of eIF6 IHC-stained material was analyzed using the Image-Pro Plus application. IOD = optical intensity of positive cells × area of positive cells. (**C,D**) Expression of eIF6 protein was analyzed by western blotting. Protein levels were quantified using Image-Pro Plus. Data were normalized to GAPDH levels. Each experiment was repeated three times. **Represents p < 0.01, and *represents p < 0.05.

The *in vivo* study was conducted using partial-thickness wounds on the mouse dorsum as described in Materials and Methods. In one group of 12 mice (STRETCHED), wounds were subjected to external mechanical stretching by removing a hexagonally shaped region of the skin and then suturing to introduce tension. This procedure was precisely controlled and consistent (Fig. 1A,C). In a second group of 12 mice (NON-STRETCHED), a sham operation was conducted to introduce a wound, but no skin was removed, and the cut was sutured without tension, allowing for natural skin stretching in the wound area (Fig. 1B,D). Our results show that external mechanical stretching significantly increases eIF6 expression in granulation tissue as demonstrated by immunohistological staining (Fig. 2A,B, p = 0.032) and western blotting (Fig. 2C,D, p = 0.001).

**Figure 3:**
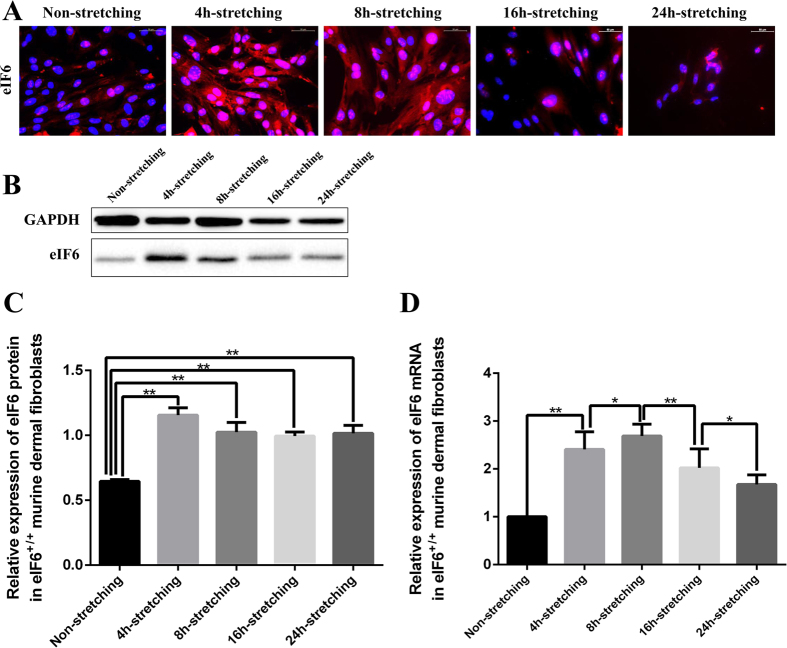
Expression of eIF6 in eIF6^+/+^ murine dermal fibroblasts subjected to external mechanical stretching. Murine dermal fibroblasts in DMEM supplemented with 10% FBS were seeded onto Bioflex^®^ six-well culture plates coated with fibronectin, and then exposed to external mechanical stretching for 4, 8, 16 and 24 h. Mechanical stretching was imposed by increasing surface area by 10% at a frequency of 0.1 Hz (6 cycles/min). (**A**) Immunofluorescence of eIF6 in eIF6^+/+^ murine dermal fibroblasts subjected to 0, 4, 8, 16 and 24 h of mechanical stretching (eIF6, red; DAPI stained nuclei, blue; 200× magnification). (**B,C**) The expression of eIF6 protein was analyzed by western blotting and quantified using Image-Pro Plus. Data were normalized to GAPDH levels. (**D**) eIF6 mRNA expression in eIF6^+/+^ murine dermal fibroblasts subjected to mechanical stretching was determined by real time RT-PCR. Expression levels in stretched cultures (gray) are displayed relative to non-stretched cultures (black; set to 1). Each experiment was conducted a minimum of three times, with similar results. Cell number did not change significantly under any conditions. **Represents p < 0.01, and *represents p < 0.05.

For the *in vitro* study, murine dermal fibroblasts were cultured in Bioflex^®^ six-well culture plates and subjected to cyclic external mechanical stretching for 0, 4, 8, 16 and 24 h at a frequency of 0.1 Hz (6 cycles/min), with a maximal increase in surface area of 10%. Cell samples were then prepared for examination by immunofluorescence. Positive staining of eIF6 is observed in the nucleus and cytoplasm of wild type (eIF6^+/+^) murine dermal fibroblasts. Cells subjected to four hours of mechanical stretching exhibit an increase in eIF6 expression, especially in the cytoplasm. However, after 16 to 24 hours of stretching, eIF6 fluorescence intensity decreases (Fig. 3A). To assess eIF6 protein expression more quantitatively, we measured eIF6 levels in stretched and non-stretched murine dermal fibroblasts using western blotting. eIF6 protein expression is increased in stretched cells over non-stretched cells at all time points, with peak levels at 4 h (Fig. 3B,C, p = 0.0002). We also measured eIF6 mRNA expression using real time RT-PCR. eIF6 mRNA expression increases as cells are stretched over 8 h, then slowly decreases. mRNA levels are greater in stretched cells over non-stretched cells at all time points, with peak levels at 8 h. eIF6 mRNA expression increases from 2.41-fold (4 h) to 2.68-fold (8 h). Levels are significantly different when non-stretched and stretched cells are compared at 4, 8, 16, and 24 h (p = 0.0014), and between cells stretched for 4 and 8 h (p = 0.00227) (Fig. 3D).

### The effect of eIF6 on murine dermal fibroblasts subjected to mechanical stretching

**Figure 4:**
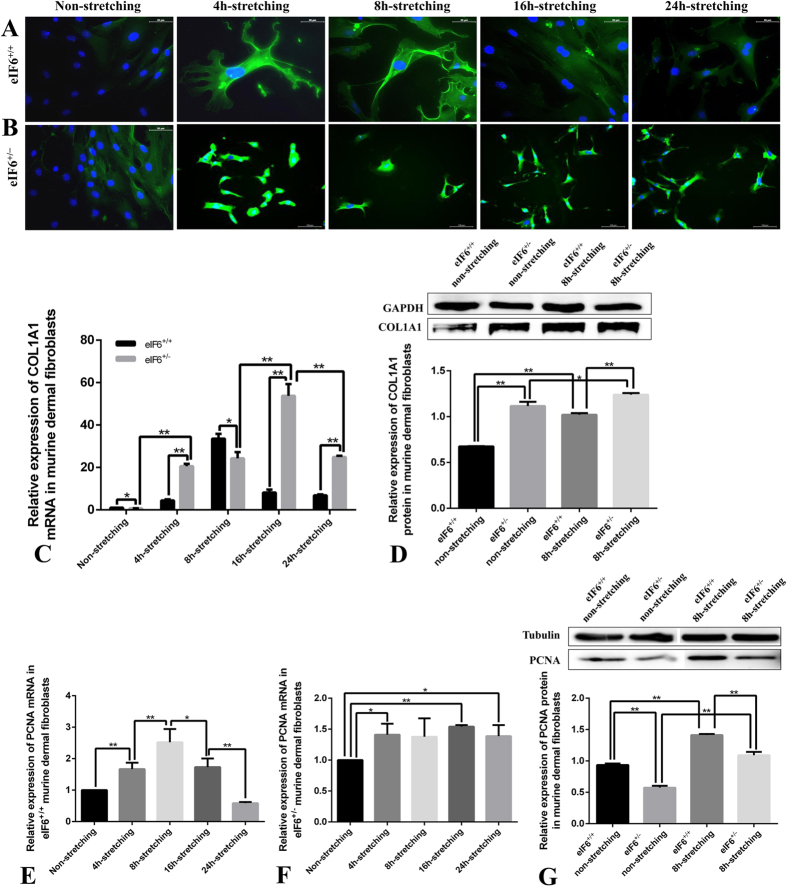
Expression of COL1A1 and PCNA in murine dermal fibroblasts subjected to external mechanical stretching. Murine dermal fibroblasts in DMEM supplemented with 10% FBS were seeded onto Bioflex^®^ six-well culture plates coated with fibronectin, and then subjected to external mechanical stretching for 0, 4, 8, 16 and 24 h. Mechanical stretching was imposed by increasing surface area by 10% at a frequency of 0.1 Hz (6 cycles /min). (**A**) Immunofluorescence of COL1A1 in eIF6^+/+^ murine dermal fibroblasts (COL1A1, green; DAPI stained nuclei, blue; 400× magnification). (**B**) Immunofluorescence of COL1A1 in eIF6^+/−^ murine dermal fibroblasts (COL1A1, green; DAPI stained nuclei, blue; 200× magnification). (**C**) Detection of COL1A1 mRNA expression by real time PCR in eIF6^+/−^ and wild type murine dermal fibroblasts. (**D**) Western blot of total COL1A1 protein in cells with or without 8 h external mechanical stretching, the data were normalized to GAPDH levels. (**E**) Detection of PCNA mRNA expression by real time PCR in wild type murine dermal fibroblasts. (**F**) Detection of PCNA mRNA expression by real time PCR in eIF6^+/−^ murine dermal fibroblasts. (**G**) Western blot of total PCNA protein in cells with or without 8 h external mechanical stretching. Data were normalized to tubulin levels. Each experiment was conducted at least three times, with similar results. Cell number did not change significantly in any conditions. Protein levels in stretched cultures (gray) are displayed relative to non-stretched cultures (black; set to 1). **Represents p < 0.01, and *represents p < 0.05.

To determine whether eIF6 is involved in regulating collagen I expression and proliferation under conditions of external mechanical stretching, we used immunofluorescence staining to compare the expression of COL1A1 and PCNA in wild type (eIF6^+/+^) and eIF6 deficient (eIF6^+/−^) murine dermal fibroblasts. COL1A1 staining is observed in the cytoplasm of ~4% of unstretched eIF6^+/+^ fibroblasts. After external mechanical stretching, COL1A1 expression is considerably altered, with 87% and 92% of cells expressing COL1A1 after 4 hours and 8 hours respectively. After 8 hours COL1A1 levels steadily decreased (Fig. 4A). In contrast, ~72% of unstretched eIF6^+/−^ fibroblasts of had detectable COL1A1 fluorescence. Stretching increased this level to 100% (Fig. 4B).

To quantitatively measure the expression of COL1A1, mRNA and protein expression in eIF6^+/+^ and eIF6^+/−^ murine dermal fibroblasts were compared using real time RT-PCR and western blotting. Cells were either non-stretched, or subjected to 8 hours of external mechanical stretching. Stretching increased COL1A1 mRNA expression in eIF6^+/+^ cells 4.46-fold at 4 h, 33.55-fold at 8 h, 8.16-fold at 16 h and 6.86-fold at 24 h over levels in non-stretched cells (Fig. 4C). Levels were significantly different in stretched vs. non-stretched cells at all time points, as well as between cells stretched for 4 and 8 h (p = 0.021), and between cells stretched for 8 and 16 h (p = 0.003). COL1A1 mRNA expression in non-stretched eIF6^+/−^ murine dermal fibroblasts was significantly lower than in eIF6^+/+^ cells (Fig. 4C). However, when subjected to external mechanical stretching, COL1A1 mRNA expression levels in eIF6^+/−^ murine dermal fibroblasts increased from 20.59-fold at 4 h to 24.28-fold at 8 h (p = 0.0002), and to 53.78-fold at 16 h (Fig. 4C, p < 0.0001). Subsequently, levels decreased to 24.91-fold at 24 h (Fig. 4C, p = 0.0006). External mechanical stretching significantly increased COL1A1 protein expression in both eIF6^+/+^ and eIF6^+/−^ cells; from 0.675-fold to 1.021 fold in eIF6^+/+^ cells (p < 0.0001), and from 1.115-fold to 1.240-fold in eIF6^+/−^cells (p = 0.0285). Values were normalized to GAPDH (Fig. 4D).

To explore the possible role of eIF6 in the proliferation of murine dermal fibroblasts in response to external mechanical stretching, the expression of PCNA, a marker of cell proliferation, was measured in eIF6^+/+^ and eIF6^+/−^ cells. In wild type fibroblasts, mRNA expression of PCNA increased during 8 hours of stretching, then decreased thereafter (Fig. 4E). In eIF6^+/−^ fibroblasts, PCNA mRNA expression increased 30–50% under stretching conditions, these elevated levels were maintained throughout the experiment (Fig. 4F). However, in comparison with eIF6^+/+^ cells, eIF6^+/−^ cells showed less proliferative capacity with or without external mechanical stretching (Fig. 4G).

### The effect of eIF6 on wound healing under external mechanical stretching

**Figure 5:**
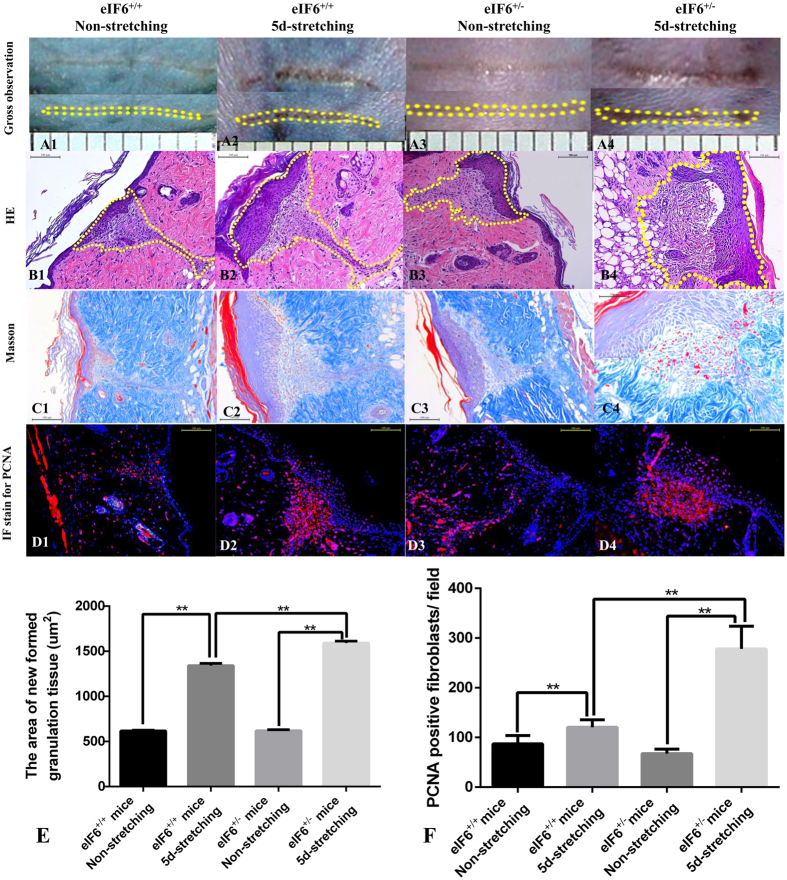
Histology of newly formed granulation tissue in partial thickness wounds in eIF6^+/+^ and eIF6^+/−^ mice. (A1–4) Gross observation of wounds. The yellow dotted lines outline the partial thickness wound area. (B1–4) HE staining of newly formed granulation tissue shown in A1–A4. (C1–4) Masson staining of newly formed granulation tissue shown in A1–A4. (D1~4) Immunofluorescence (IF) staining for PCNA in newly formed granulation tissue. The areas occupied by newly formed granulation tissue in the partial-thickness wound (**E**) and immunofluorescence-stained PCNA (**F**) were quantified. **Represents P < 0.01 and *represents P < 0.05. (n = 4).

To further elucidate the effects of eIF6 on wound healing under conditions of external mechanical stretching, we examined the expression of COL1A1 and PCNA in partial-thickness wounds *in vivo*. Newly formed granulation tissue as well as collagen deposition, increased significantly under external mechanical stretching in eIF6^+/−^ and eIF6^+/+^ mice (Fig. 5). However, more granulation tissue formed in eIF6^+/−^ mice than in eIF6^+/+^ mice (Fig. 5E, p < 0.0001). External mechanical stretching also increased the number of PCNA positive cells, compared to levels in non-stretched animals (Fig. 5D,F). This effect was greater in eIF6^+/−^ cells than in eIF6^+/+^ cells (Fig. 5F, p = 0.0005).

### Effect of eIF6 on the TGF-β/Smad pathway under external mechanical stretching

**Figure 6:**
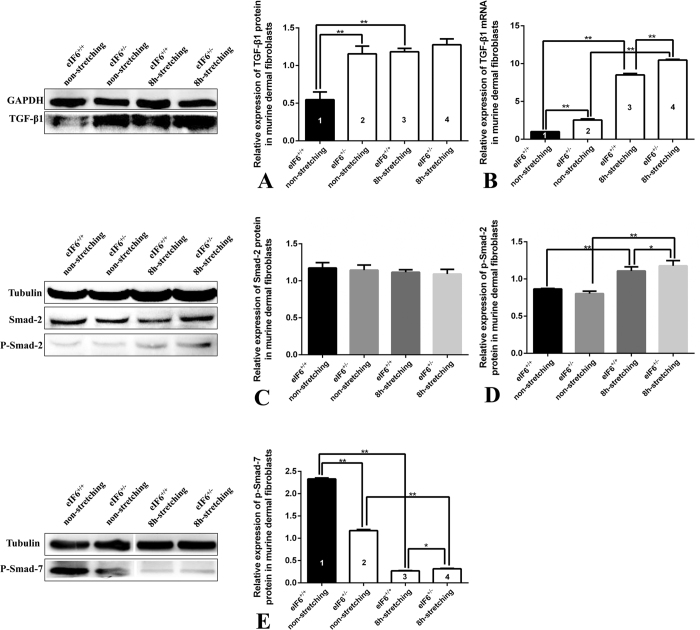
Expression of key proteins in the TGF-β/Smad pathway in eIF6^+/+^ and eIF6^+/−^ murine dermal fibroblasts subjected to external mechanical stretching. (**A**) TGF-β1 protein expression in murine dermal fibroblasts (eIF6^+/+^ and eIF6^+/−^) with or without 8 hours external mechanical stretching. The data were normalized to GAPDH levels. (**B**) TGF-β1 mRNA expression in murine dermal fibroblasts (eIF6^+/+^ and eIF6^+/−^) with or without 8 hours external mechanical stretching. Protein levels in stretched cultures are displayed relative to non-stretched cultures (black; set to 1). Each experiment consisted of four replicates for each condition. (**C**) Smad2 protein expression. The data were normalized to tubulin levels. (**D**) p-Smad2 expression. (**E**) p-Smad7 protein expression. The data were normalized to tubulin levels. Each experiment was repeated three times; data shown are representative. **Represents p < 0.01 and *represents p < 0.05.

To explore the possible involvement of the TGF-β/Smad pathway in eIF6-mediated dermal fibroblast function, we used real time RT-PCR and western blotting to measure mRNA and protein expression levels of TGF-β1, Smad2, p-Smad2 and p-Smad7 in eIF6^+/+^ and eIF6^+/−^ murine dermal fibroblasts subjected to 8 h of mechanical stretching. The results showed that eIF6 deficiency increased the expression of TGF-β1 protein (Fig. 6A, bar 1 vs. bar 2) and mRNA (Fig. 6B, bar 1 vs. bar 2). Although the TGF-β1 mRNA expression levels in eIF6^+/+^ and eIF6^+/−^ cells were significantly different, both for non-stretched (bar 1 vs bar 2) and stretched (bar 3 vs bar 4) cells (Fig. 6B), the magnitudes of expression were similar (1 fold vs. 2.54 fold for non-stretched cells, and 8.51 fold vs. 10.47 fold for stretched cells) (Fig. 6B). Interestingly, there was no significance difference in TGF-β1 protein levels in eIF6^+/+^ and eIF6^+/−^ cells subjected to stretching (Fig. 6A, bar 3 vs. bar 4).

Intracellular signaling mediated by TGF-β1 was examined as well. Smad2 protein levels did not change significantly in eIF6^+/+^ or eIF6^+/−^ cells subjected to stretching, compared to non-stretched cells (Fig. 6C). However, p-Smad2 levels increased significantly in both cell types after 8 hours of stretching (Fig. 6D). Smad7, which plays an inhibitory role in the TGF-β/Smad pathway, was expressed at significantly lower levels in eIF6^+/−^ cells (Fig. 6E, bar 1 vs. bar 2, p < 0.0001). In the presence of external mechanical stretching, p-Smad7 levels further decreased (Fig. 6E, bars 3 and 4 vs. bars 1 and 2). p-Smad7 levels were significantly different in eIF6^+/+^ and eIF6^+/−^ cells subjected to stretching (p = 0.0015) (Fig. 6E, bars 3 and 4).

### Possible role of TGFβR I/II in eIF6-mediated dermal fibroblast function under external mechanical force

**Figure 7:**
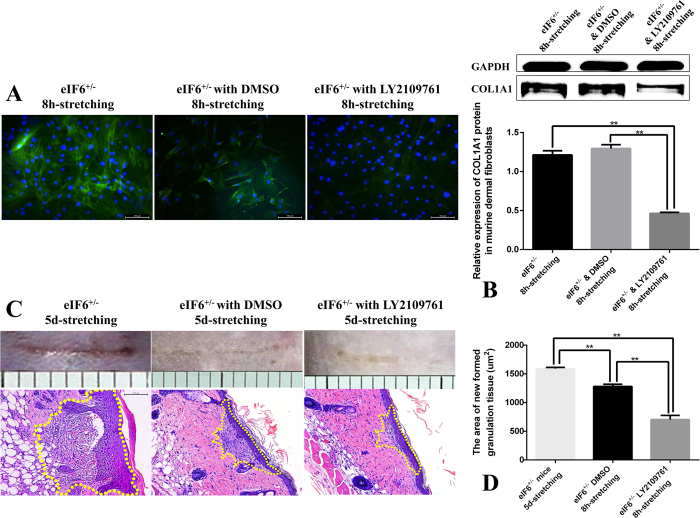
Possible role of TGFβR I/II in eIF6-mediated dermal fibroblast function under external mechanical force. (**A**) Immunofluorescence staining of COL1A1 for eIF6^+/−^ murine dermal fibroblasts (COL1A1, green; DAPI depicts nuclei, blue; 200× magnification). (**B**) Western blotting of COL1A1 protein of murine dermal fibroblasts with or without LY2109761 after 8 hours external mechanical stretch. Each experiment was conducted a minimum of three times, with similar results. Cell number was not significantly changed under all conditions. (**C**) The newly-formed granulation in model of partial-thickness wound of eIF6^+/−^ mice were subcutaneously injected with TGFβR I/II inhibitor LY2109761 and exposed to 5-day mechanical stretching. (**D**) The area of newly formed granulation tissue in the model of partial-thickness wound (n = 4). The data was morphologically quantified using Image-Pro Plus software. **Represents p < 0.01 levels of significance.

The data described above show that the increased COL1A1 expression observed in eIF6^+/−^ cells could be increased further by external mechanical stretching, suggesting that mechanical stretching plays a synergistic role in promoting COL1A1 expression. To better understand the possible role of TGFβR I/II in eIF6^+/−^ murine dermal fibroblasts under these conditions, we used LY2109761 (a dual inhibitor for both TGF-β receptor type I and type II) to block TGFβR I/II in eIF6^+/−^ murine dermal fibroblasts, and then observed collagen I expression levels after 8 hours of external mechanical stretching. LY2109761 significantly suppressed COL1A1 protein expression in cells subjected to stretching compared with non-stretched controls (Fig. 7A,B).

This experiment was also conducted *in vivo*. LY2109761 was injected subcutaneously in close proximity to the partial-thickness wound^19^. The results showed that LY2109761 reduced the area of granulated tissue in eIF6^+/−^ mice subjected to external mechanical stretching (Fig. 7C,D).

## Discussion

The development of fibrosis presents a great challenge to researchers^20^,^21^,^22^. The control of fibrosis remains elusive, although some progress has been made^23^,^24^,^25^. It is well known from clinical experience that retention sutures prevent fibrosis to a certain extent^26^, but the underlying mechanisms have yet to be clarified.

We recently reported that eukaryotic initiation factor 6 (eIF6), which is involved in ribosomal biogenesis and translational control by regulating the binding of the 40S and 60S ribosomal subunits, regulates fibrosis via the TGF-β1 pathway in animal models^16^, human hypertrophic scarring^15^, and chronic kidney disease^27^. eIF6 is also involved in regulating MMP-2/TIMP-2 ratios to balance the degradation and deposition of the extracellular matrix^28^. We were therefore motivated to ask whether eIF6 is involved in mechanical force-mediated fibrosis, and implemented an *in vitro* external mechanical stretch model using the Flexcell tension system. Results using this model demonstrate that eIF6 and COL1A1 expression increases in fibroblasts subjected to external mechanical stretching, and eIF6 mRNA expression increases in murine dermal fibroblasts after 4 hours of stretching (Fig. 3D). This may be an adaptive mechanism to reduce fibrotic response. Protein levels after stretching increase relatively less than the corresponding levels of mRNA in both wild type and eIF6^+/−^ murine fibroblasts. The absence of a direct correlation between mRNA and protein levels suggests that the relationship between mRNA and protein levels is not strictly linear. One possible reason is that different regulatory mechanisms (e.g., independently targeting synthesis and degradation rates), act on both mRNA and protein, affecting the abundance of the two molecules differentially. After 24 hours, eIF6 mRNA expression decreases slowly with increasing a-SMA expression (data not shown), perhaps related to the increasing differentiation of fibroblasts. In addition, eIF6 deficient cells exhibit increased COL1A1 expression when subjected to mechanical stretching, compared with eIF6 wild type cells. eIF6 may therefore be involved in the regulation of external mechanical force-mediated fibroblast function.

Because TGF-β pathway activity is intimately involved in fibrosis and collagen deposition, and eIF6 inversely modulates the expression of TGF-β1, mainly at the transcription level^16^, eIF6 deficiency may result in increased collagen I deposition. This prediction is supported by our results in which the level of TGF-β1 mRNA expression, as well as p-Smad protein expression, is increased in cells containing reduced levels of eIF6, suggesting the activation of TGF-β1signal transduction. Furthermore, a significant decrease in Smad7 protein expression was observed in eIF6 deficient cells, and even greater reductions occurred in these cells subjected to external mechanical stretching. However, there was no significant difference in TGF-β1 and Smad2 protein levels in stretched vs non-stretched eIF6 deficient cells. It is possible that eIF6 and external mechanical stretching have an additive or synergistic effect on TGF-β1 mRNA expression, but not on TGF-β1 protein expression. External mechanical stretching may impact TGF-β1 protein regulation far less than eIF6 deficiency.

We have also shown that the increased COL1A1 expression (mRNA and protein) and TGF-β1 mRNA expression in eIF6^+/−^ murine dermal fibroblasts increases further with external mechanical stretching, and that inhibition of TGFβR I/II significantly reduces COL1A1 protein expression in these cells. These results suggest that mechanical stretching plays a synergistic role in promoting COL1A1 expression in eIF6^+/−^ cells, and the increase in TGF-β1 mRNA expression stimulated by external mechanical stretching is at least partly achieved by the activation of TGFβR I/II. TGFβR I/II is of great importance in mechano-sensitive pathways^14^,^29^, supporting a role for these receptors in mechanotransduction in collaboration with eIF6. Because eIF6 levels were reduced only by 50% in our eIF6 deficiency model, we conclude that fibroblast cells are highly sensitive to perturbations of eIF6, and that eIF6 may act as a protective factor to maintain cell homeostasis under external mechanical stretching.

**Figure 8:**
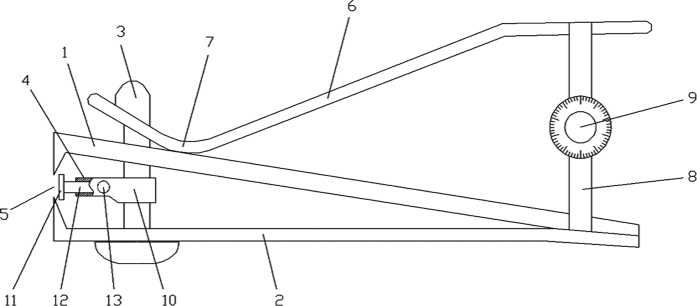
Novel tool designed to introduce partial-thickness wounds in mice. (1) Upper clamp body; (2) lower clamp body; (3) shaft; (4) depth control assembly comprised by components 10 through 13 described below; (5) clamping tong; (6) pressing lever; (7) leveraging fulcrum; (8) adjustment belt; (9) length control screw; (10) holder to secure depth control assembly; (11) barrier plate for depth control; (12) connecting rod; (13) locking screws.

Another goal of this study was to investigate the role of eIF6 in mechanical force-mediated fibrosis *in vivo*. A protocol was designed to deliver a controlled stretch stress to a partial-thickness wound (Fig. 1). The partial-thickness wound was generated using an apparatus consisting of an upper and lower body clamp, a depth control, a pressure-activated handle, an adjustable belt and a length control screw (Fig. 8). The tool accurately controlled lesion area as well as wound depth. Average wound depth was 0.50 ± 0.072 (data not shown). Using this device, we demonstrated that external mechanical stretching induced granulation tissue formation, and eIF6^+/−^ mice exhibited significantly more granulation tissue formation than wild type mice. In contrast, TGFβR I/II inhibition significantly reduced granulation tissue formation under external mechanical stretching. These results provide convincing evidence that eIF6, TGFβR I/II activation and mechano-sensitive pathways are linked *in vivo*. In addition, this data strongly supports our previous report that eIF6 expression is extremely low or absent in the basal layer of the epidermis early in hypertrophic scar formation, and then increases slowly as scar formation proceeds^15^. These stages parallel the development of mechanical loading, which is often lower at earlier stages of healing but increases with the development of granulation tissue^30^.

## Conclusion

The experiments presented here reveal a possible mechanism for external mechanical force-mediated dermal fibroblast function and fibrosis. The results show that the increased mechanical load in a wound bed causes a significant increase in eIF6 expression. This response may protect normal dermal fibroblasts from collagen over-production partly through the regulation of the TGF-β/Smad pathway.

## Materials and Methods

### Cell culture and external mechanical stretching

Primary murine dermal fibroblasts were isolated from the skin of wild type (eIF6^+/+^) and eIF6 heterozygous (eIF6 deficient, eIF6^+/−^) newborn C57BL/6 mice (purchased from Fondazione Centro San Raffaele del Monte Tabor, Italy) using standard procedures. The eIF6 gene was deleted by homologous recombination using embryonic stem cell technology. eIF6^+/−^ murine fibroblast cells have a 50% reduction in eIF6 levels, but retain sufficient nucleolar eIF6 and exhibit normal ribosome biogenesis^31^. All cells were initially cultured in Dulbecco’s Modified Eagle’s Medium (DMEM) (Sigma-Aldrich, St. Louis, MO, USA) supplemented with 10% Fetal Bovine Serum (FBS, Sigma-Aldrich, catalog No. 12303C) and penicillin (100 IU/mL)–streptomycin (100 μg/mL). 0.5 × 10^6^ cells in a total volume of 10 ml were seeded into the wells of Bioflex^®^ six-well culture plates coated with 10 mg/ml of fibronectin (BF-001P, Flexcell, USA), which promotes cell attachment to the silicone surface without affecting fibroblasts differentiation^32^,^33^.

One day after cell seeding, cultures were assigned to the STRETCHED or NON-STRETCHED groups. The STRETCHED group was further subdivided into the “mechanical stretch group”, “mechanical stretch & LY2109761 group”, and “mechanical stretch & dimethyl sulfoxide (DMSO) group”. The “mechanical stretch group” was further divided into four subgroups that were subjected to stretching for 4, 8, 16 and 24 h. We choose 8 h as the observation time point for the “mechanical stretch & LY2109761 group” and “mechanical stretch & DMSO group”, for which the mRNA expression of target proteins had reached their peak, and thus the changes in protein expression might be more significant. LY2109761 (a dual inhibitor for both TGF-β receptor type I and type II, Selleck, Houston, TX) was dissolved in 100% dimethyl sulfoxide (DMSO) at a stock concentration of 10 mmol/L, and the concentration of DMSO did not exceed 0.1% in any assay. Before tests, all culture medium was replaced with fresh medium. The experiments were conducted using an FX-5000T Flexercell Tension Plus (Flexcell International Corporation, Hillsborough, NC, USA). Cells were subjected to cyclic tension for 4, 8, 16 and 24 h at a frequency of 0.1 Hz (6 cycles/min), with a maximal increase in surface area of 10%.

### Animal model and external mechanical stretching

Pure breed C57BL/6 male mice (wild type, eIF6^+/+^, purchased from the Third Military Medical University, China) and eIF6 heterozygous male mice (eIF6 deficient, eIF6^+/−^, purchased from Fondazione Centro San Raffaele del Monte Tabor, Italy) aged 8~10-week weighing 20~22 g were backcrossed to C57BL/6 mice as described in the literature^16^. Mice were anesthetized with an intraperitoneal injection of 1% (w/v) sodium pentobarbital (0.01 ml/g body weight). After shaving the dorsal hair and cleaning the exposed skin with 75% ethanol, the partial-thickness wounds were created on the dorsum of the mice. To establish a stable animal model, we designed and constructed a mouse partial-thickness wound tool (Fig. 8). To introduce a wound, the tool was pressed vertically onto the mouse back, and a lever was pressed to create the wound. Lesion area and wound depth were both reproducibly adjustable. Wounded mice were assigned to the STRETCHED or NON-STRETCHED group, and subjected to either “natural skin stretching” (5 days, n = 12 mice) or “external mechanical stretching” (5 days, n = 12 mice). “Natural skin stretching” refers to a constant mechanical environment in the vicinity of the partial thickness wound after a sham surgical operation, followed by tension-free suturing. “External mechanical stretching” refers to the persistent mechanical stretching of the partial-thickness wound, imposed by tension suturing of a hexagonally shaped wound. We selected 5 days after injury as an observation point for the STRETCHED group, because wound inflammation begins to decline 5 days after injury, and the proliferation of fibroblasts increases along with the generation of granulation tissue. To examine the role of TGFβR I/II in mechano-transduction, the wounds in the STRETCHED group were further divided into 3 subgroups designated the “mechanical stretch group”, “mechanical stretch & LY2109761 group”, and “mechanical stretch & DMSO group” (n = 4 mice per group). LY2109761 was dissolved in 100% DMSO at a stock concentration of 10 mmol/L, and the concentration of DMSO did not exceed 0.1% in any assay. After the experiment, wounds were examined for the induction of granulation tissue. For expression analyses, one wound sample from each animal was frozen in liquid nitrogen immediately after excision. All experimental methods described above were conducted in accordance with guidelines for animal care and were approved by the First Affiliated Hospital (Southwest Hospital) of the Third Military Medical University. All experiments were approved by the Laboratory Animal Welfare and Ethics Committee of the Third Military Medical University.

### Histopathology

Partial thickness wounds were dissected and immediately fixed in 4% paraformaldehyde in 0.1 M PBS at pH 7.4 for at least 48 h, then embedded in paraffin. Successive transverse paraffin sections were cut at ~4 um thickness and were subjected to HE staining as well as Masson’s trichrome staining.

### Immunohistochemical analysis

Immunohistochemistry was performed on formalin-fixed, paraffin-embedded tissue as well as on cells growing on Bioflex^®^ six-well culture plates. Briefly, sections or cell culture plates were deparaffinized in xylene and rehydrated in a series of increasingly dilute ethanol solutions. All specimens were quenched with 3% hydrogen peroxide (H_2_O_2_) to block endogenous peroxidase, and pretreated by microwave heating for 10 min in antigen unmasking solution (pH = 6.8, 0.1 M citrate buffer, Zhongshan Jinqiao Biology Corporation, Beijing) to increase staining. To block non-specific background, the sections or slides were blocked with 3% bovine serum albumin (BSA) for 30 min. Specimens were incubated with antibodies for eIF6 (1:400, CST, #3263, USA) at 4 °C overnight, followed by the appropriate secondary antibodies. Signals were amplified with 3,3-diaminobenzidine and counterstained with hematoxylin. Finally, specimens were dehydrated and mounted using cover slips. For all samples, negative controls were incubated with 0.1 M PBS as a substitute for the primary antibody.

Digital images of each section were captured with a Leica Confocal Microscope (Leica Microsystems, Wetzlar, Germany) at 200x magnification. Images were randomly obtained from five fields for each sample. Expression intensity of positively labeled cells was measured and analyzed using the Image-Pro^®^ Plus 6.0 application (Media Cybernetics, USA). Data were expressed as the integral optical density (IOD) (IOD = optical intensity of positive cells × area of positive cells).

### Immunofluorescence analysis

Murine dermal fibroblast cells and wound tissue sections were rinsed with phosphate-buffered saline (PBS), immediately fixed in 4% paraformaldehyde in 0.1 M PBS at pH 7.4 at room temperature, and processed for immunofluorescence microscopy. Fibroblast cell samples were permeabilized with Triton X-100, and tissue samples were sectioned and incubated with proteinase K (Millipore, Bedford, MA) for 20 min at 37 °C.

To detect PCNA expression in newly formed granulation tissue, each sample was incubated at 4 °C for at least 16 h with the primary antibody (anti-PCNA 1:400, CST, #13110, USA) diluted in blocking solution, followed by incubation with Alexa Fluor secondary antibody at a dilution of 1:200 for 1 h. Nuclei were stained with DAPI for 10 min after incubation with the secondary antibody.

To detect the expression of eIF6 and COL1A1 in dermal fibroblasts, the stretchable films, along with the attached fibroblast cells, were severed from the Bioflex^®^ six-well culture plates and incubated at 4 °C for at least 16 h with the primary antibodies (anti-eIF6 1:400, CST, #3263, USA; anti-COL1A1 1:200, abcam, ab34710, UK) diluted in blocking solution, followed by incubation with Alexa Fluor secondary antibody at a dilution of 1:200 for 1 h. Samples were mounted on blank slides with an anti fluorescence quenching agent.

All images were acquired using fluorescence microscopy. Digital images were captured with a Leica Confocal Microscope (Leica Microsystems, Wetzlar, Germany) at 400× or 200× magnification and were randomly obtained from five fields each section.

### Western blotting

Total protein from each murine dermal sample (from which the epidermal layer had been removed) was extracted with lysis buffer (50 mmol/L Tris HCl pH 7.5; 2% sodium deoxycholate) and quantified using the bicinchoninic acid assay (Pierce, USA). Equal amounts of protein extracted from each sample were subjected to SDS-polyacrylamide gel (12%) electrophoresis, followed by transfer to polyvinylidene fluoride membranes (Millipore). All gels were run under identical conditions. Membranes were blocked in 3% bovine serum albumin (BSA) in Tris-buffered saline containing 0.1% Tween 20 (TBST) for 1 hour to prevent nonspecific adsorption. Primary antibody reactions were performed with antibodies against eIF6 (CST, #3263, USA), TGF-β1 (CST, #3711, USA), PCNA (CST, #13110, USA), COL1A1(abcam, ab34710, UK), Smad2 (abcam, ab63576, UK), p-Smad2 (abcam, ab188334, UK) and p-Smad7 (abcam, ab11392, UK) at a 1:2000 dilution with 3% BSA in TBST. Glyceraldehyde-3-phosphate dehydrogenase (GAPDH, abcam, ab8245, UK) or Tubulin (abcam, ab6046, UK) were used for normalization of data, (primary antibodies for both were diluted 1:5000 with 3% BSA in TBST). After extensive washing, immunoreactive bands were visualized using Enhanced Chemiluminescence (ECL, Amersham Biosciences) Western blotting detection substrate, imaged with the ChemiDoc^TM^ XRS+(Bio-Rad, Hercules, CA, USA) detection system, and then analyzed by densitometry using the Image J image analysis package (National Institutes of Health, Bethesda, MD). The data are shown as average values calculated from a minimum of three individual experiments. Error bars represent the standard deviation from the average value.

### Real time PCR (qRT-PCR)

Total RNA was extracted from BSA-gradient purified fibroblast cells using RNeasy Mini kits (Qiagen, Valencia, CA), reversed transcribed using superscript II single-strand synthesis reverse transcriptase [RT] (Invitrogen), and subjected to real-time PCR with SYBR green I (Toyobo) in a 7500 Real-Time PCR System (Applied Biosystems). mRNA levels for eIF6, PCNA, TGF-β1 and COL1A1 were measured using the following primers:

eIF6 (forward: 5′-AGAGCGTCGTTCGAGAACAAC-3′

reverse: 3′-CGGGAATGGCATCGGAGAG-5′);

COL1A1 (forward: 5′-TTCTCCTGGCAAAGACGGACTCAA-3′

reverse: 3′-AGGAAGCTGAAGTCATAACCGCCA-5′);

PCNA (forward: 5′-CACCTTAGCACTAGTATTCGAAGCAC- 3′

reverse: 3′-CATTATTTCTACGGCAGCCCAC-5′);

TGF-β1 (forward: 5′-CCGCAACAACGCCATCTATG-3′

reverse: 3′-CTCTGCACG GGACAGCAAT-5′);

GAPDH (forward: 5′-CGTGCCGCCTGGAGAAAC-3′

reverse: 3′-AGTGGGAGTTGCTGTTGAAGTC-5′.

Amplification was performed as follows: initial denaturation for 5 min at 95 °C, 40 cycles of denaturation at 95 °C for 15 s, annealing at 60 °C for 15 s, and extension at 72 °C for 20 s. Reaction products were quantified using GAPDH expression as a reference, and values were plotted as relative transcript abundance.

### Statistical analysis

All data were expressed as mean ± SD and statistically compared using Dunnett’s multiple-comparison tests as well as Paired samples T-tests (two-tailed). Post-hoc tests were conducted using the GraphPad Prism application. Statistical significance is indicated in figures as *P < 0.05 and **P < 0.01 where appropriate.

## Additional Information

**How to cite this article**: Shu, Q. *et al*. Involvement of eIF6 in external mechanical stretch–mediated murine dermal fibroblast function via TGF-β1 pathway. *Sci. Rep.* **6**, 36075; doi: 10.1038/srep36075 (2016).

**Publisher’s note:** Springer Nature remains neutral with regard to jurisdictional claims in published maps and institutional affiliations.

## Supplementary Material

Supplementary Information
