# Supplementary material for: Involvement of eIF6 in external mechanical stretch–mediated murine dermal fibroblast function via TGF-β1 pathway
Source: Sci Rep. 2016 Nov 8;6:36075. doi: 10.1038/srep36075 (PMC5099925; doi:10.1038/srep36075)

**Involvement of eIF6 in external mechanical stretch−mediated murine dermal fibroblast function via TGF−β1 pathway**

Qin Shu^1,2^, Jianglin Tan^1,3^, Van Daele Ulrike^4^, Xiaorong Zhang^1,3^, Jiacai Yang^1,5^, Sisi Yang^1,3^, Xiaohong Hu^1,3^, Weifeng He^1,3^, Gaoxing Luo^1,3*^, Jun Wu^1,3*^

1 Institute of Burn Research, State Key Laboratory of Trauma, Burn and Combined Injury, Southwest Hospital, The Third Military Medical University, Chongqing, 400038, People’s Republic of China.

2 Nursing Department, The Third Military Medical University, Chongqing, 400038, People’s Republic of China. 3 Chongqing Key Laboratory for Disease Proteomics, The Third Military Medical University, Chongqing, 400038, China.

4 Department of Rehabilitation Sciences and Physiotherapy, University of Antwerp, Belgium.

5 Department of Urology, Second Affiliated Hospital, Third Military Medical University, Chongqing, 400037, People's Republic of China.

* Corresponding Authors (Jun Wu: junwupro@126.com; Gaoxing Luo: logxw@yahoo.com)

**Supplementary information**

**Full-length gels and blots for figure 2**


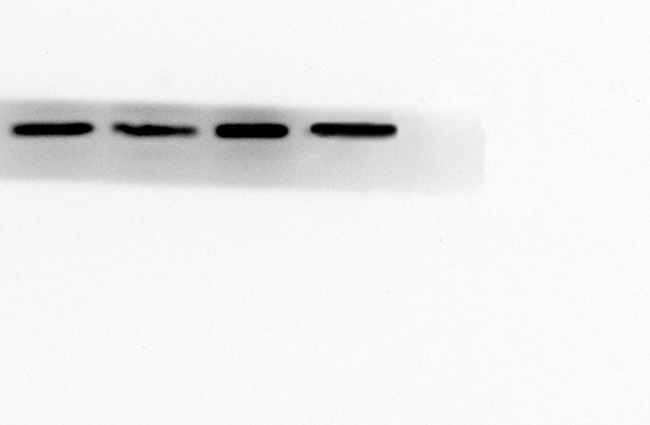

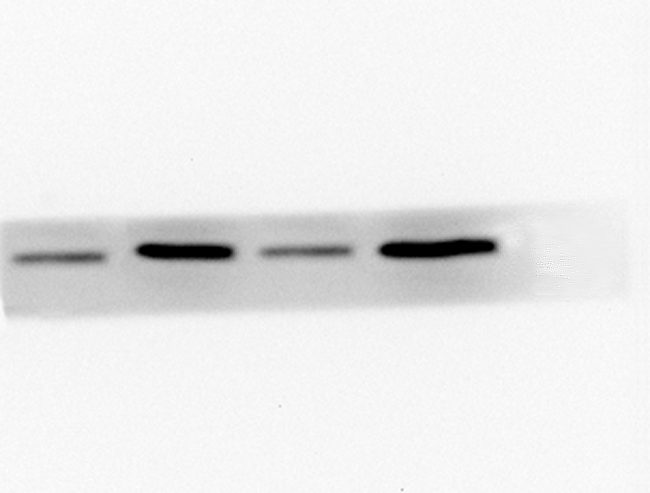


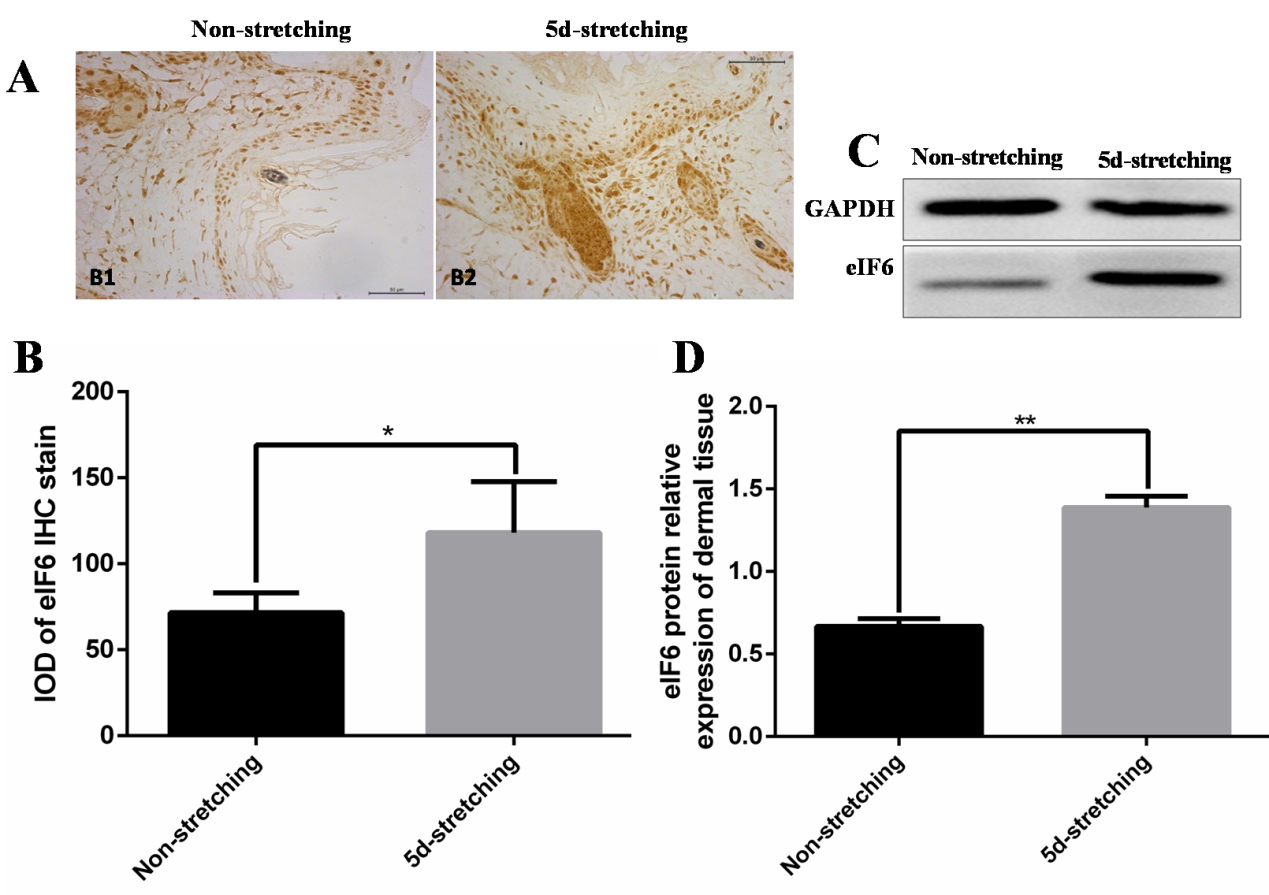


**Full-length gels and blots for figure 3**


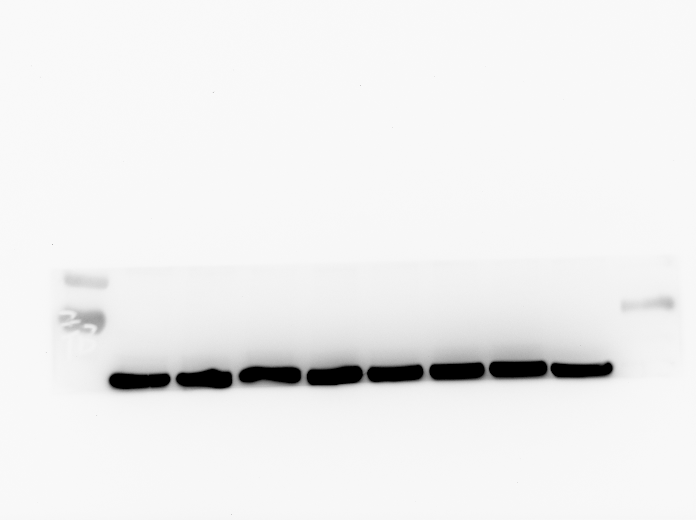

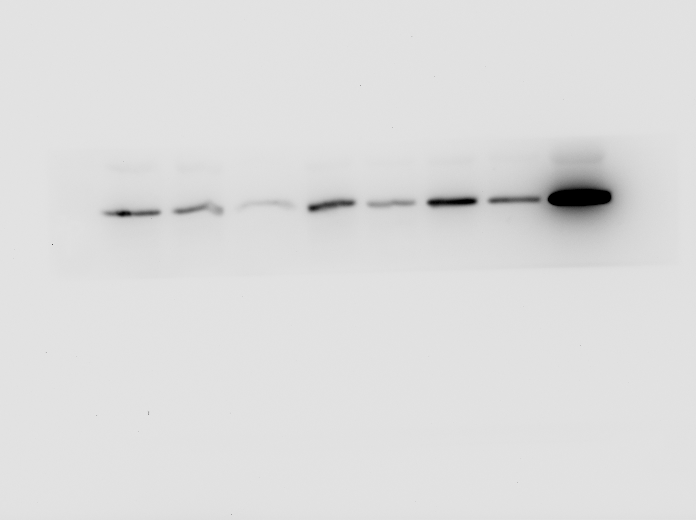


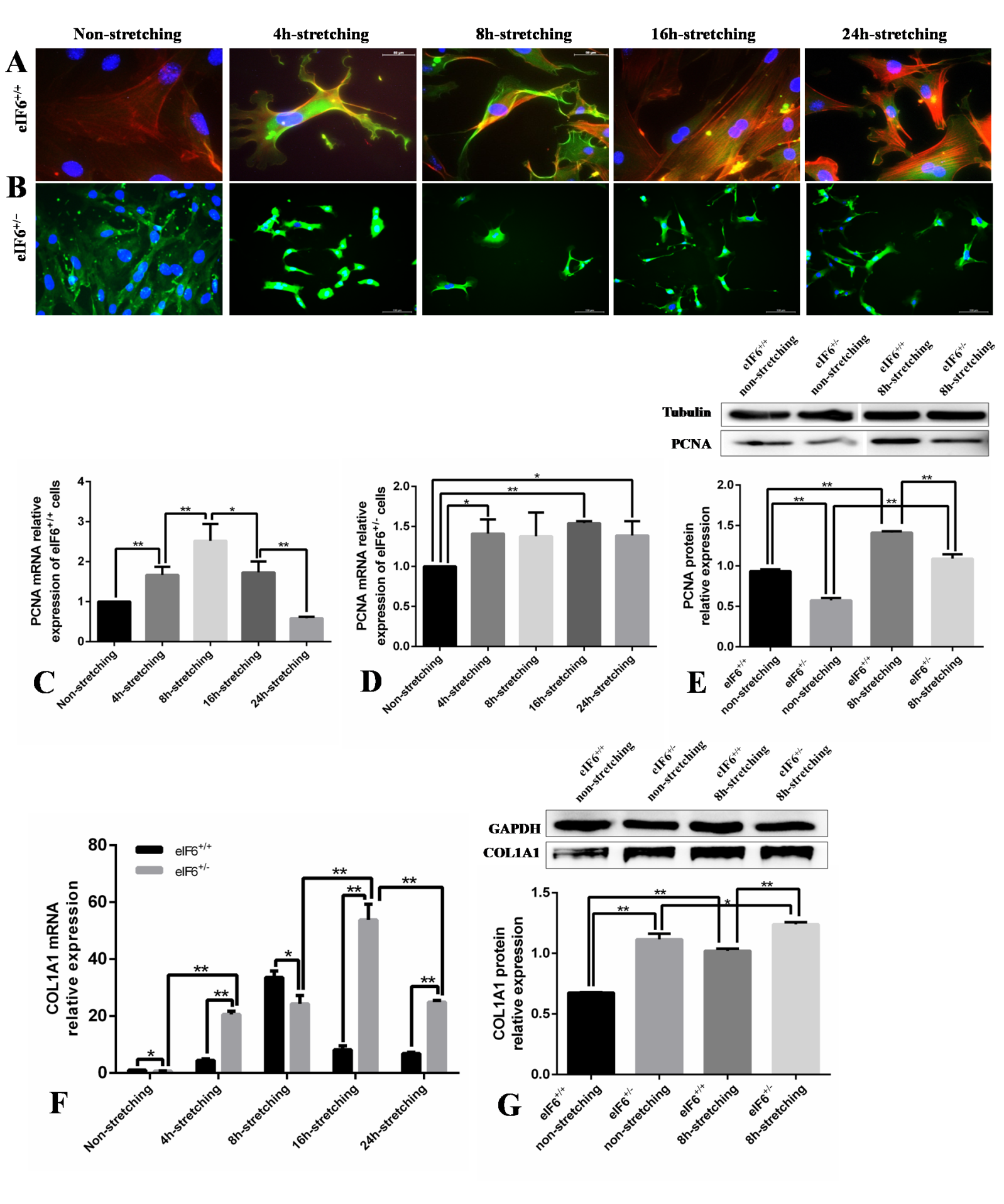


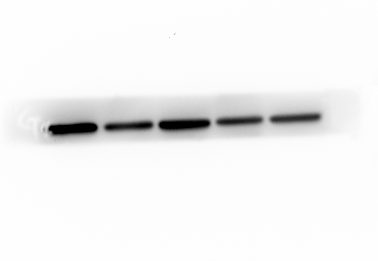

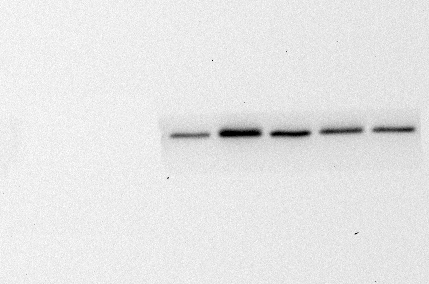


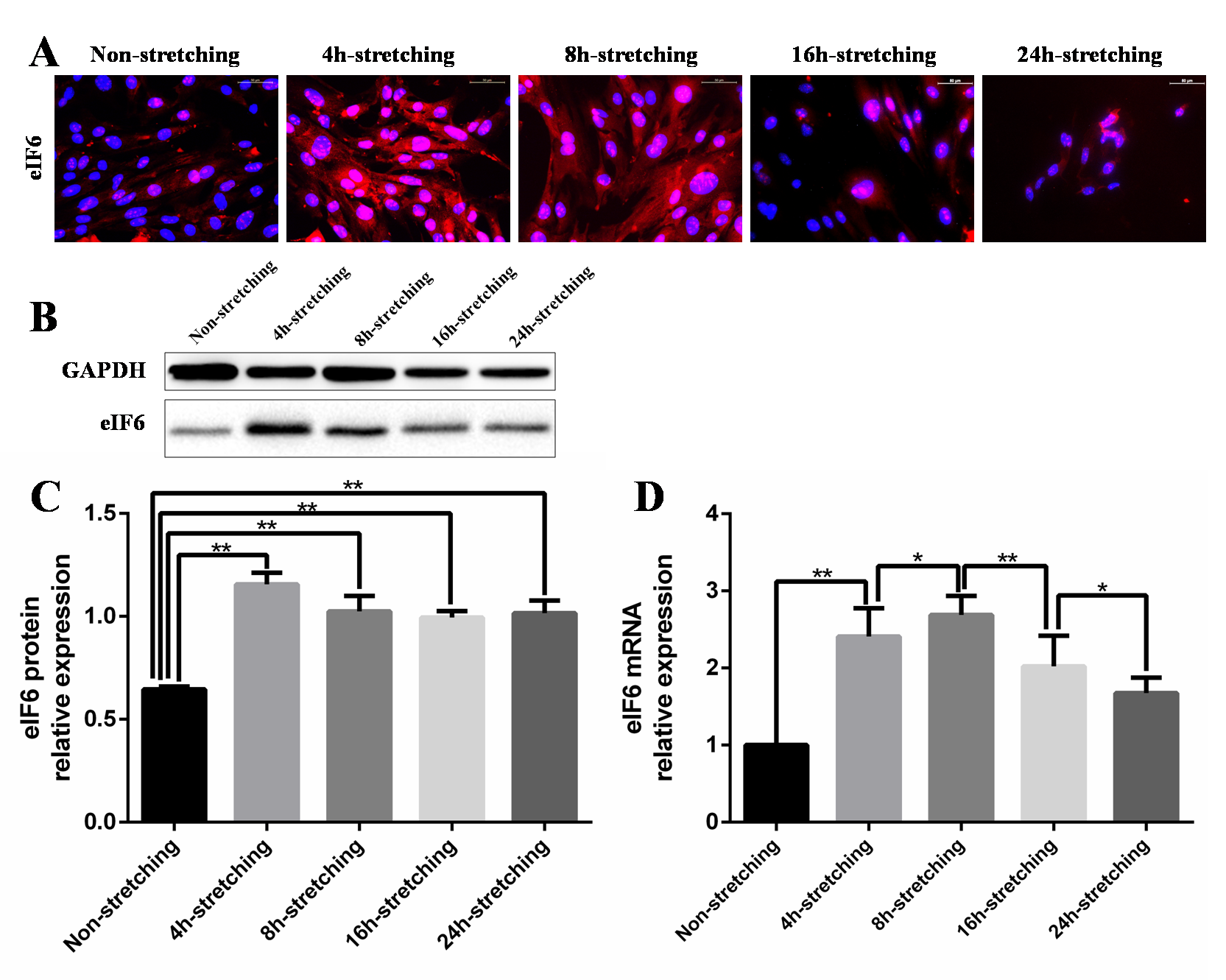


**Full-length gels and blots for figure 4**


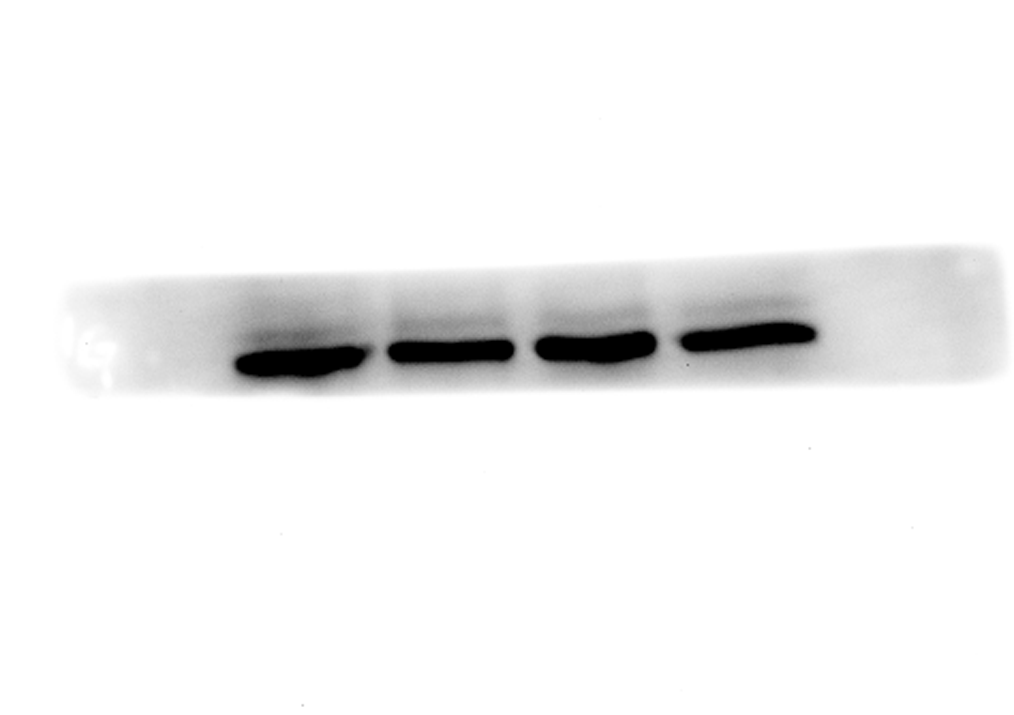

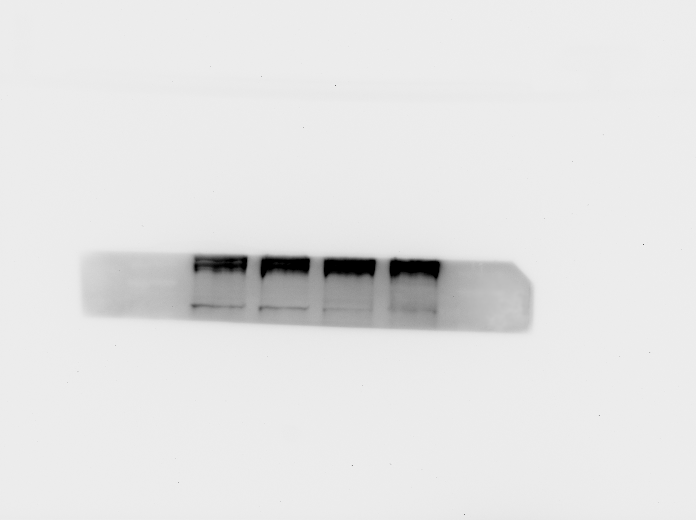


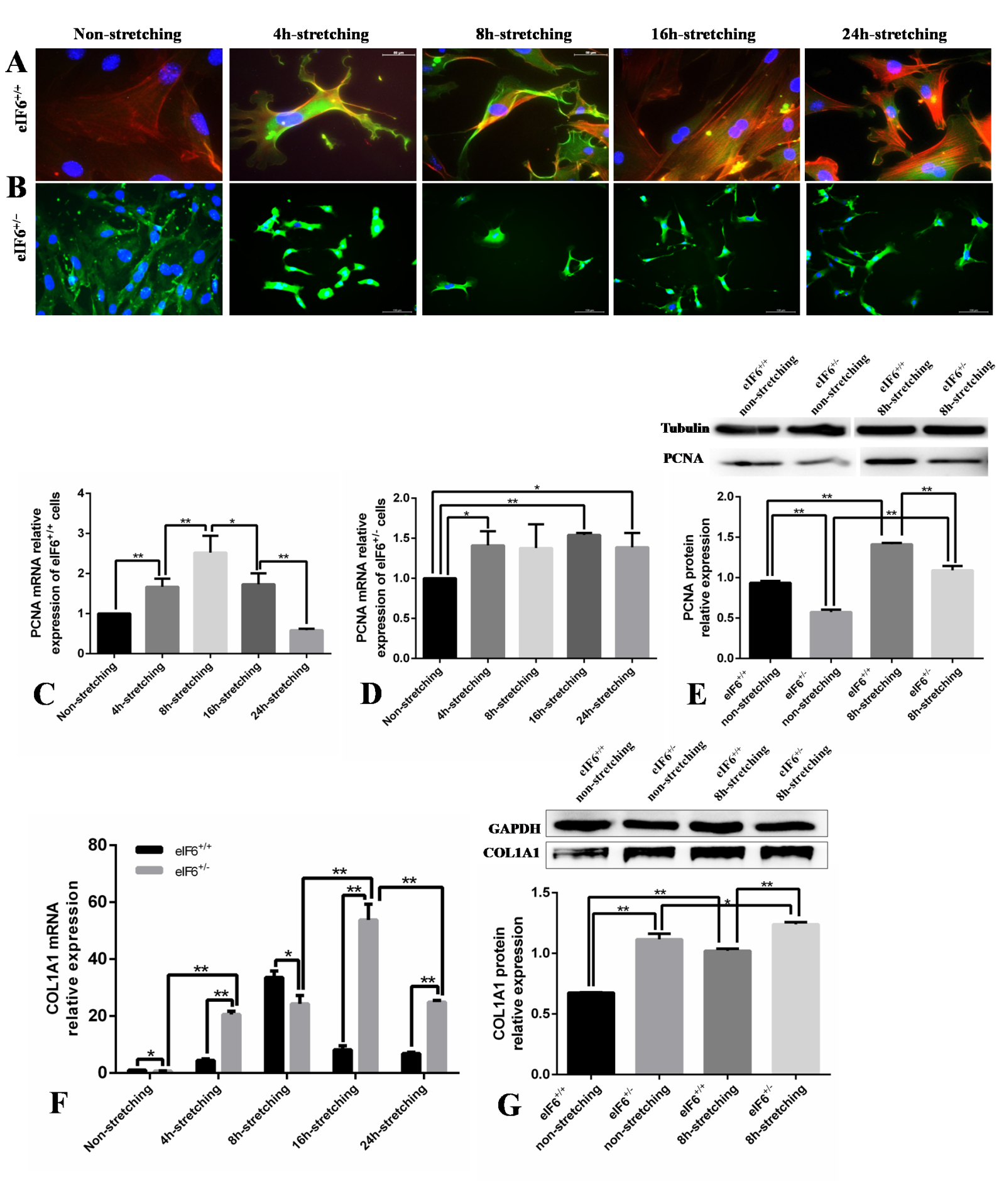


**Full-length gels and blots for figure 6**


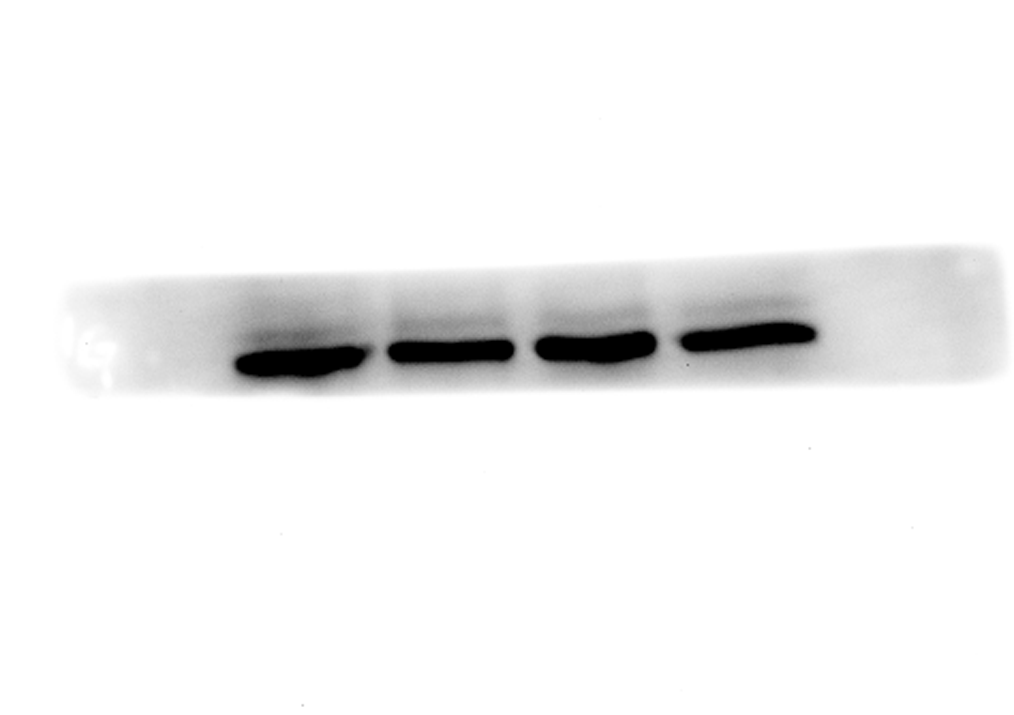

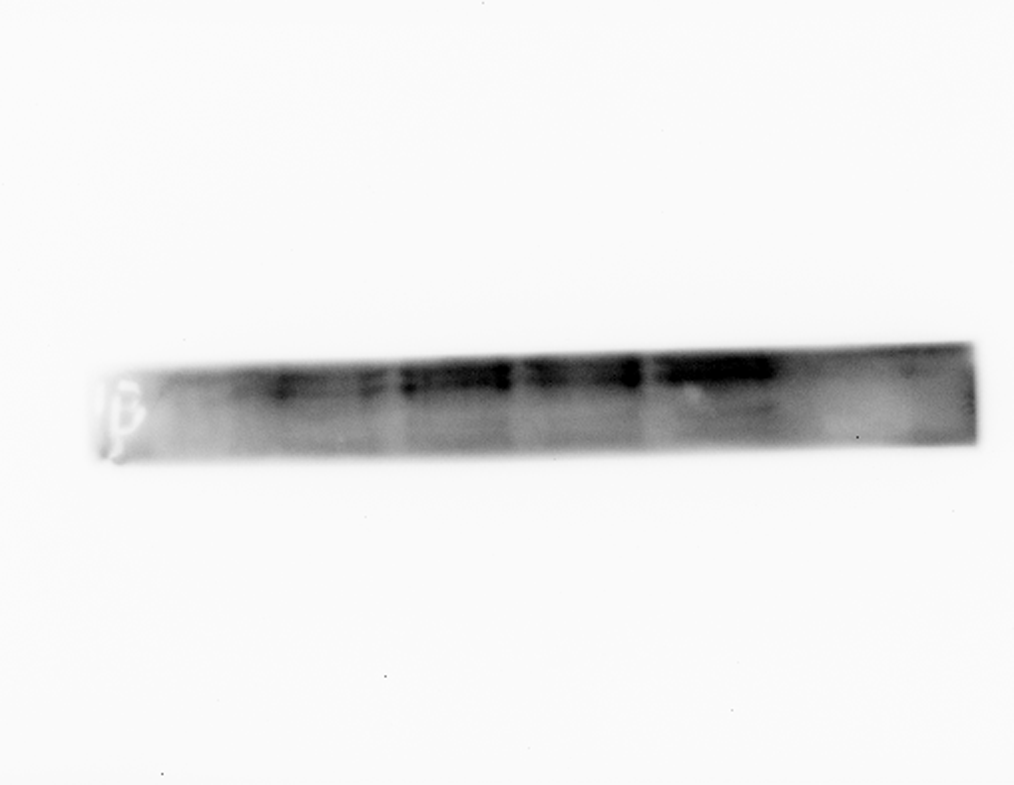


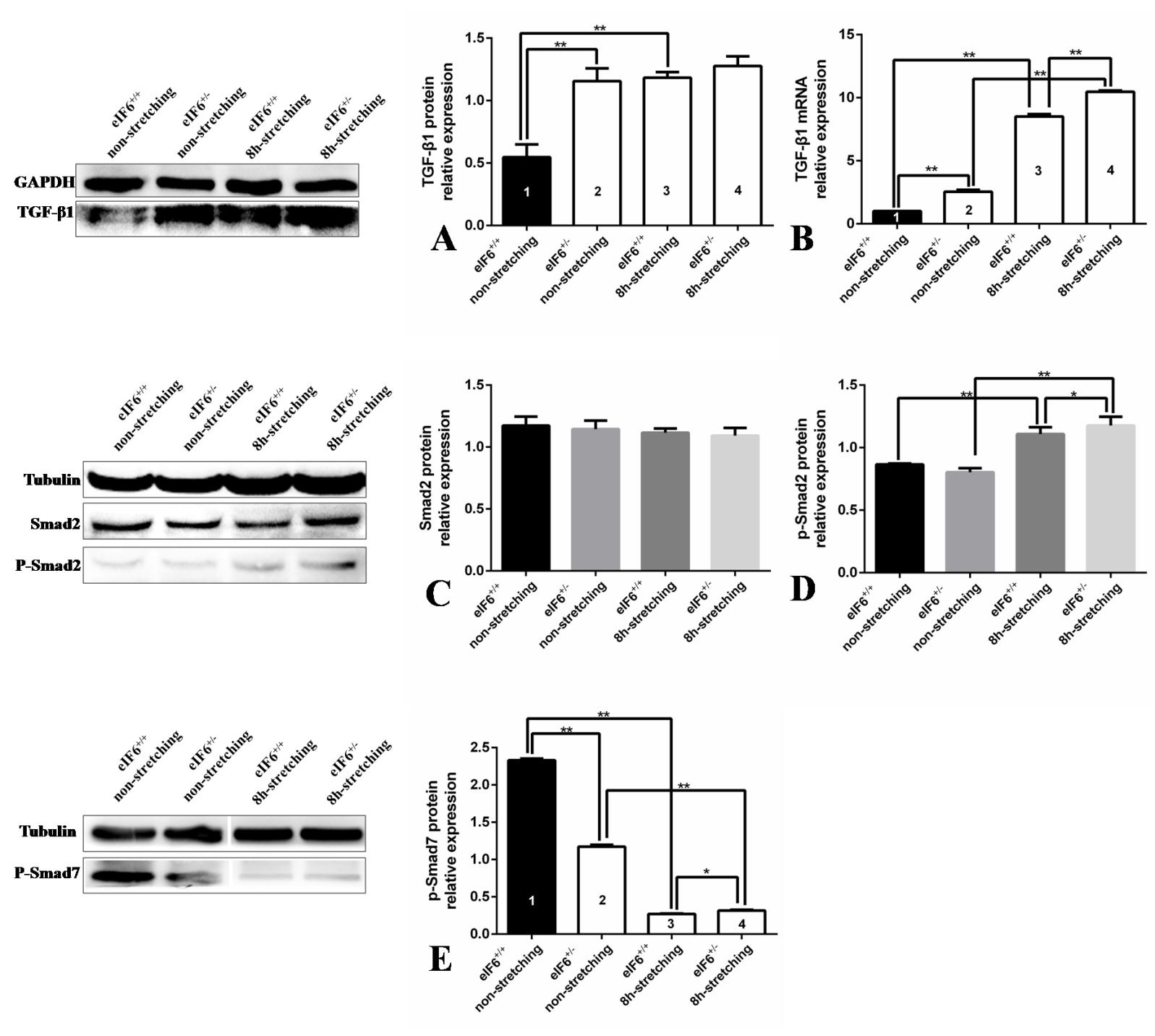


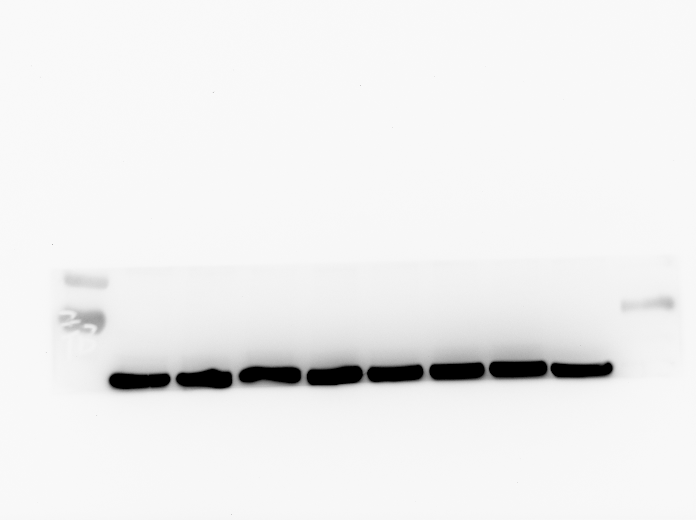

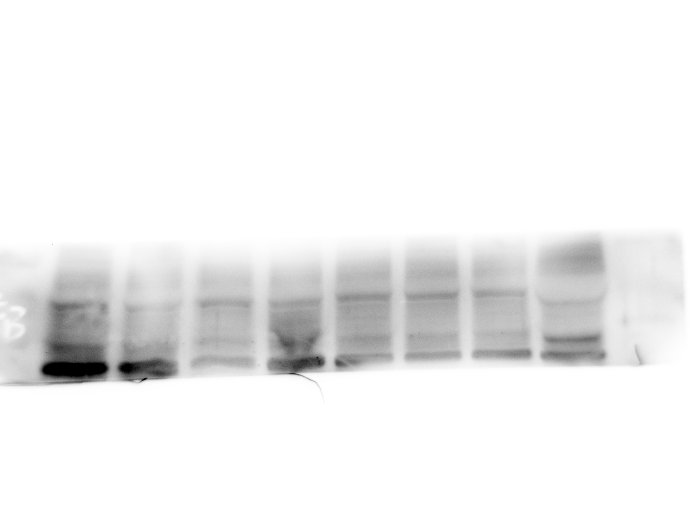


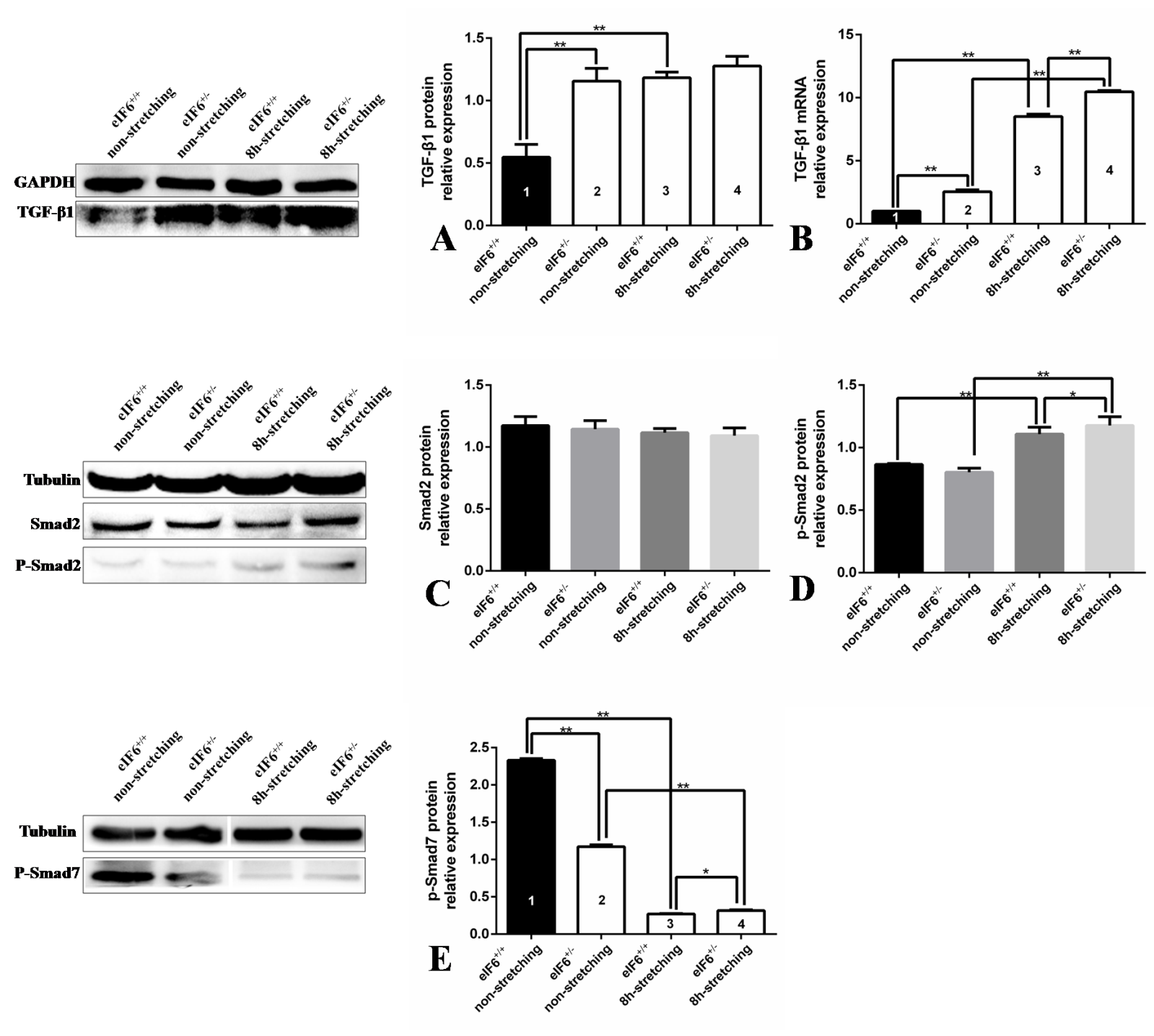


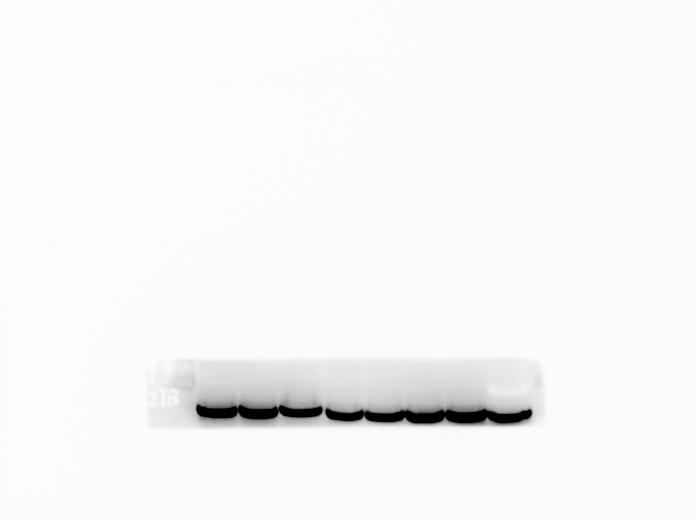

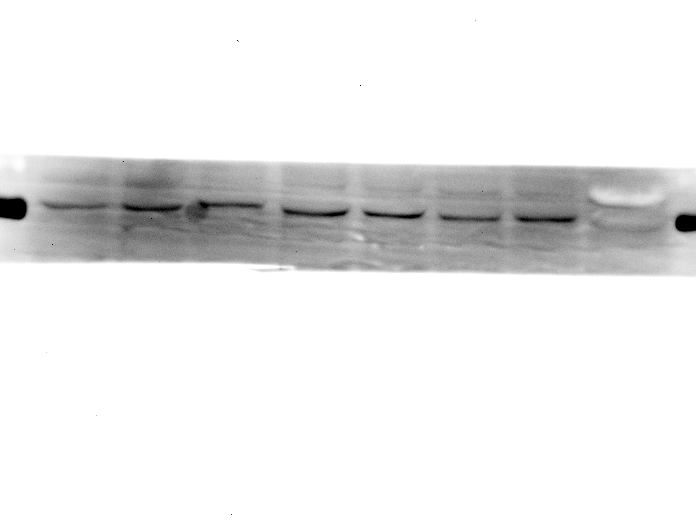

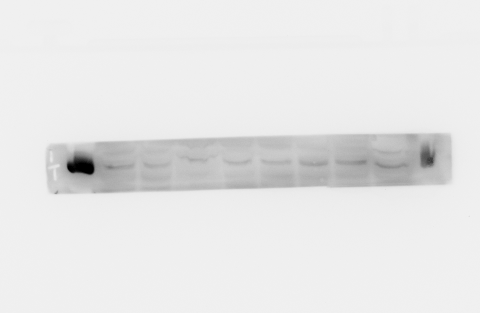


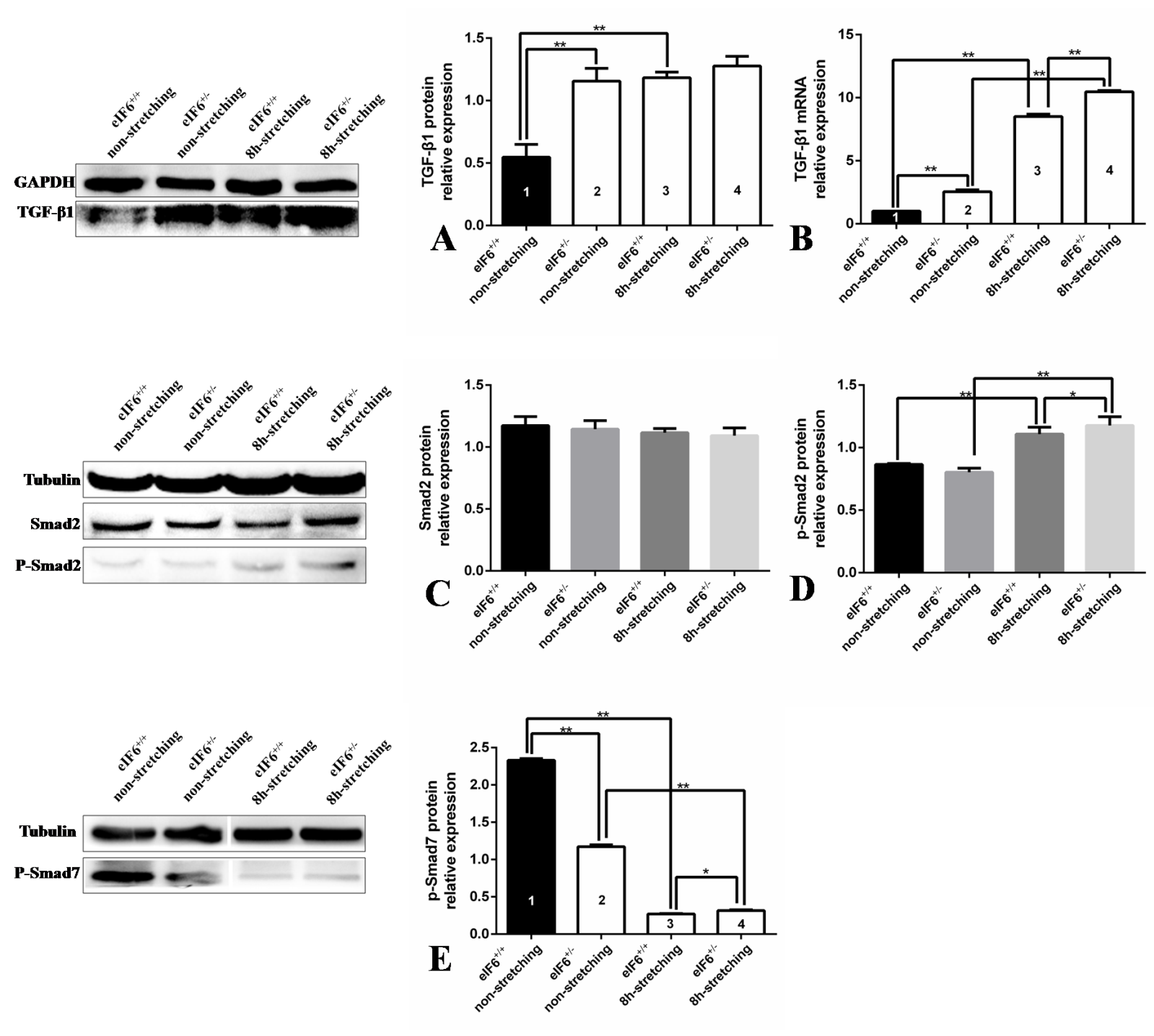


**Full-length gels and blots for figure 7**


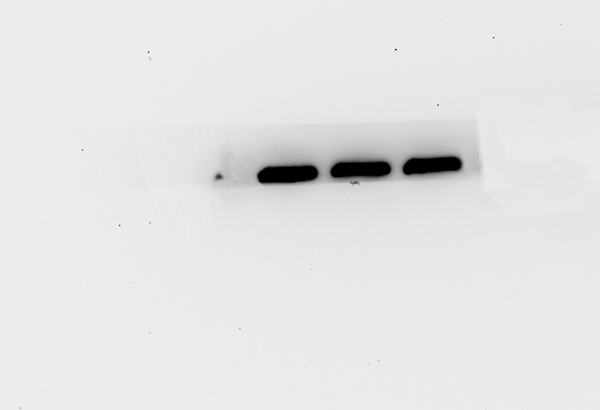

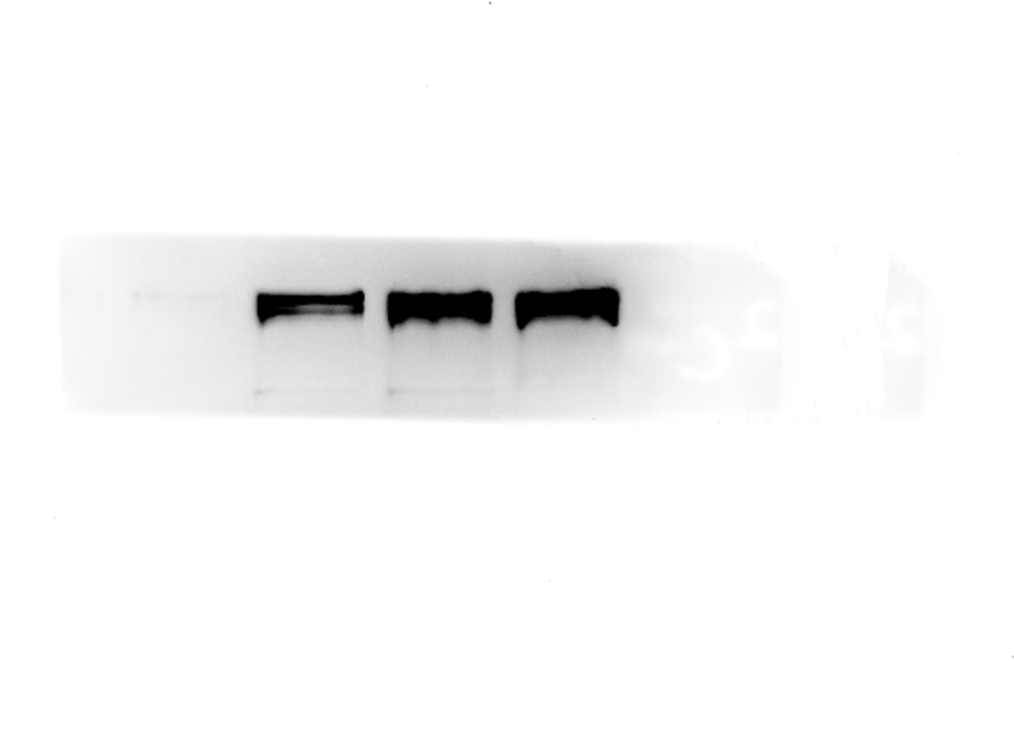


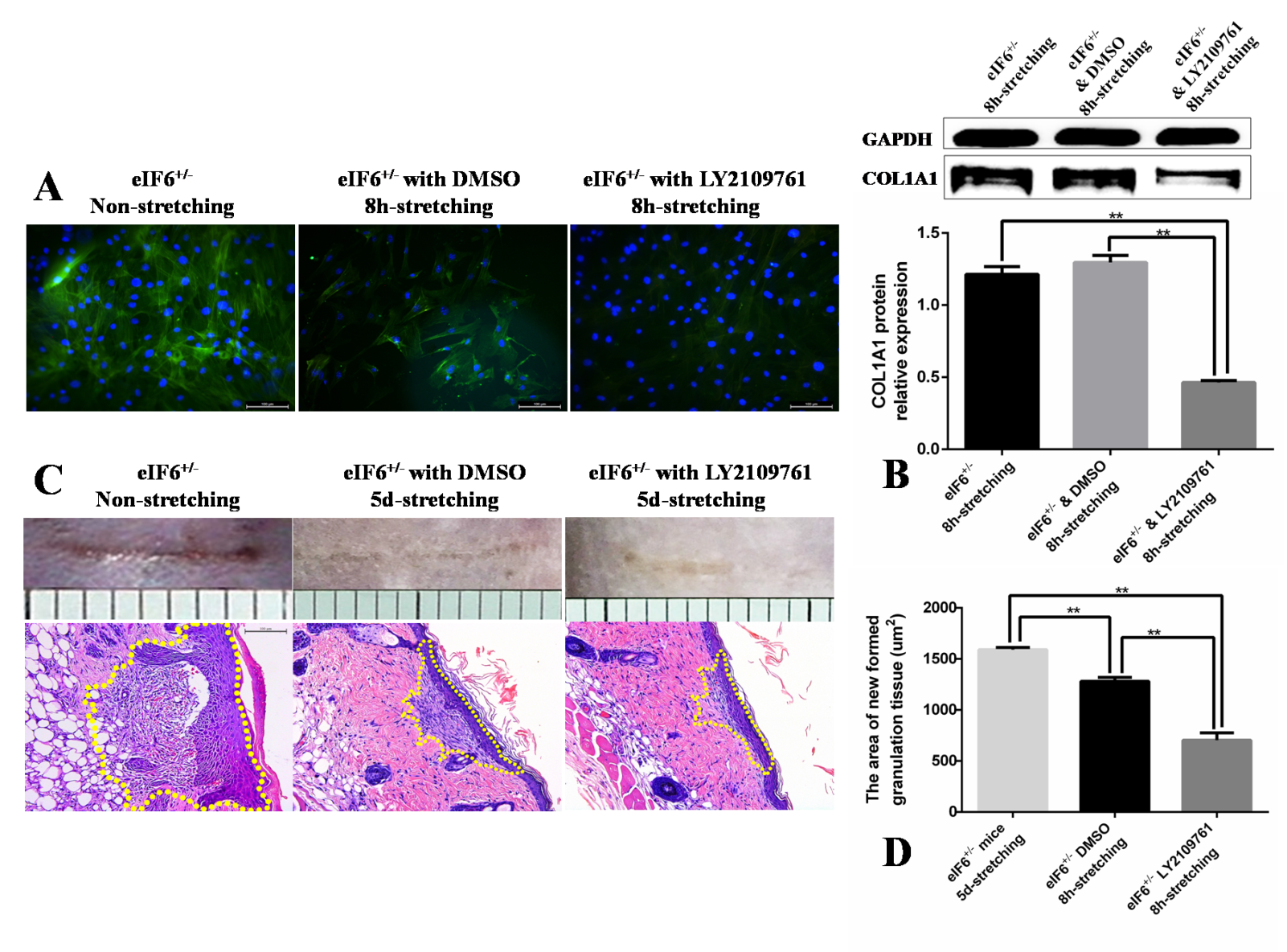

Supplement: Supplementary Information [file srep36075-s1.doc]
